# Effect on survey response rate of hand written versus printed signature on a covering letter: randomised controlled trial [ISRCTN67566265]

**DOI:** 10.1186/1472-6963-5-52

**Published:** 2005-08-09

**Authors:** Kirstie McKenzie-McHarg, Lucy Tully, Simon Gates, Sarah Ayers, Peter Brocklehurst

**Affiliations:** 1 National Perinatal Epidemiology Unit University of Oxford Old Road Campus Oxford OX3 LF UK

## Abstract

**Background:**

It is important that response rates to postal surveys are as high as possible to ensure that the results are representative and to maximise statistical power. Previous research has suggested that any personalisation of approach helps to improve the response rate. This experiment tested whether personalising questionnaires by hand signing the covering letter improved the response rate compared with a non-personalised group where the investigator's signature on the covering letter was scanned into the document and printed.

**Methods:**

Randomised controlled trial. Questionnaires about surgical techniques of caesarean section were mailed to 3,799 Members and Fellows of the Royal College of Obstetricians and Gynaecologists resident in the UK. Individuals were randomly allocated to receive a covering letter with either a computer printed signature or a hand written signature. Two reminders were sent to non-respondents. The outcome measures were the proportion of questionnaires returned and their time to return.

**Results:**

The response rate was 79.1% (1506/1905) in the hand-signed group and 78.4% (1484/1894) in the scanned and printed signature group. There was no detectable difference between the groups in response rate or time taken to respond.

**Conclusion:**

No advantage was detected to hand signing the covering letter accompanying a postal questionnaire to health professionals.

## Background

Large surveys of clinical practice are often conducted by postal questionnaire, as this is a practical and economical method of obtaining information. However, if the response rate is low, the respondents may represent a biased sample of the population. It is therefore desirable that the response rate to a postal survey be as high as possible, to ensure that there is sufficient statistical power and the findings are representative. One factor that may influence response rate is whether the covering letter that is sent out with the questionnaire is hand signed or bears a photocopied or printed signature. The rationale is that individuals who receive a hand signed covering letter may feel that the letter is more personalised to them, and hence may be more likely to complete and return the questionnaire than those who receive a letter with a printed signature.

A recent systematic review [1] included a meta-analysis of randomised controlled trials investigating the effects of personalisation of covering letters accompanying postal questionnaires. The results suggested that an approach in the form of personalised letters, questionnaires or envelopes improved final response rates by a modest amount (odds ratio 1.16, 95% confidence interval (1.07, 1.26)). However, various methods of personalisation such as hand written versus typed salutations and hand written versus typed postscripts were used by the studies in this analysis. Only five of the 48 studies in this analysis evaluated the effects of hand signing letters versus duplicated or printed signatures, and none of these involved a questionnaire sent to health professionals. These studies also had methodological problems such as low response rates and small sample sizes. None has suggested any major advantage to one group or the other [2-6].

The randomised controlled trial reported here aimed to determine whether a hand written or computer printed signature on the covering letter influenced the proportion of questionnaires returned or the time taken to return them, in a survey of clinical practice among obstetricians and gynaecologists in the UK.

## Methods

### Setting and participants

This study was conducted as part of a national postal survey of surgical techniques used in caesarean section operations in the UK [7]. The survey questionnaire was sent to all Members and Fellows of the Royal College of Obstetrician and Gynaecologists (RCOG) resident in the UK. The three page questionnaire, comprising 27 questions, asked for information about obstetricians' usual technique for lower segment caesarean section operations. Addresses were obtained from a database supplied by the RCOG. This contained 3,969 names and addresses, of which 170 were ineligible to receive the survey questionnaire; 169 had an address not in the UK, and one was an investigator for this study (PB). 3,799 questionnaires were therefore sent out.

### Interventions

The 3,799 survey recipients were randomly allocated (using a random number generator) to receive the covering letter accompanying the questionnaire signed by hand or bearing a computer printed signature. In the hand signed group, all letters were signed by the same investigator (PB) in blue ink. In the computer printed group, PB's signature was scanned into a computer file, which was imported into each letter and printed along with the letter in black ink. All letters had a personal salutation, and the letters were identical apart from the signature.

Each recipient received a copy of the questionnaire, the covering letter and a return white envelope labelled with a FREEPOST address. The survey was sent out in February 1999. Two reminders were sent to non-respondents, six and ten weeks after the initial mailing. Reminder letters included computer printed or hand written signatures according to the original allocation.

The sample size of 3,799 was sufficient to detect an absolute difference in the response rate between the groups of just over 4%, with 80% power, assuming a response rate of 70% in the control group.

## Results

By October 1999, 2,990 questionnaires had been returned, giving an overall response rate of 78.7%. The response rate was 79.1% (1506/1905) for the hand signed group and 78.4% (1484/1894) for the computer printed group. These proportions were not significantly different (risk ratio 1.01, (95% confidence interval 0.98, 1.04); risk difference +0.7% (95% confidence interval -1.9%, +3.3%)).

The overall median time taken to return the questionnaire was 16 days (interquartile range 9 to 49 days). Kaplan-Meier survival analysis was performed on time to response and no difference between the groups was detected (Log rank = 0.72, p = 0.39; Figure 2). The survival curves for the two groups were very close at all time points, and hence it is unlikely that there could be an advantage to either group in the number of reminder letters needed.

## Discussion

This study was large enough to detect a difference between the intervention and control groups of 4% or larger. The results indicate that a hand-signed covering letter is unlikely to improve the response rate or time taken to respond to a postal survey when compared with a covering letter bearing a computer printed signature. However, smaller differences between the two groups cannot be excluded and there may be a small advantage to personalisation. These findings, in a group of health professionals, support those from previous randomised trials of this intervention using other groups of respondents [2-6]. Combining the results of the existing trials in a fixed effects meta-analysis gave a summary risk ratio of 1.01 (95% CI 0.98, 1.04; Figure 3).

## Conclusion

The findings suggest that there may be no or only a very small advantage to hand signing of covering letters, and future postal surveys could use covering letters with scanned and printed signatures without compromising the response rate. However, other methods of personalising covering letters (such as handwritten postscripts or salutations) may be more beneficial in terms of enhancing response rates to questionnaires.

## Competing interests

The author(s) declare that they have no competing interests.

## Authors' contributions

PB was involved in study design and delivered the intervention. SG was involved in study design, conducted the analysis and helped to draft the manuscript. KM was involved in study design, data management and helped to draft the manuscript. SA was involved in study design, data management and analysis. LT was involved in the analysis. All authors read and approved the final manuscript.

**Figure 1:**
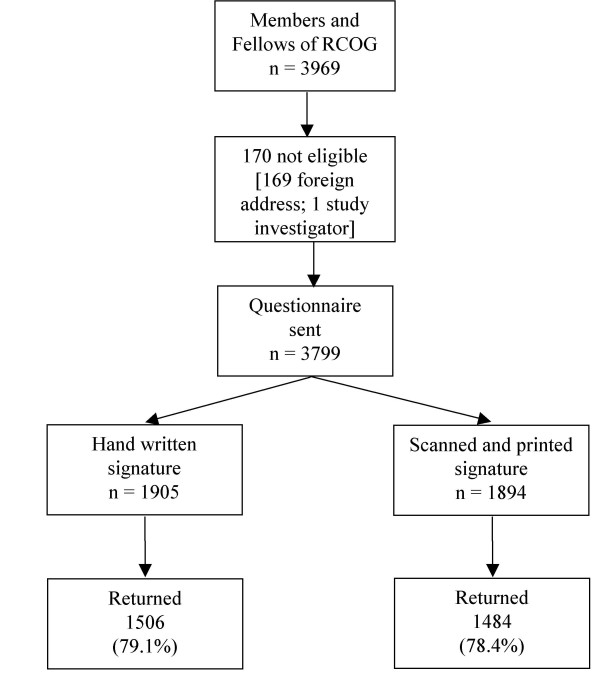
Flow chart of study participants.

**Figure 2:**
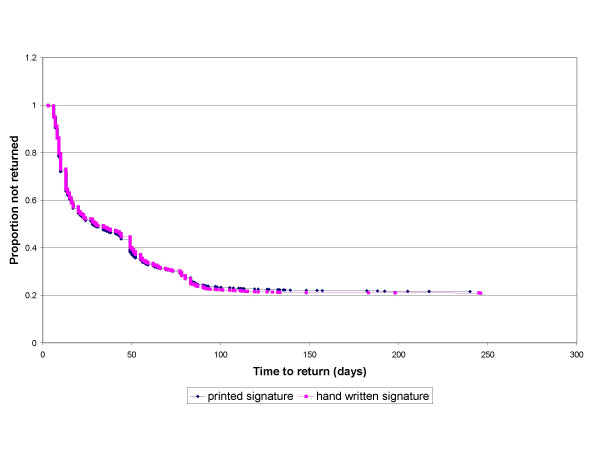
Time to return of questionnaires.

**Figure 3:**
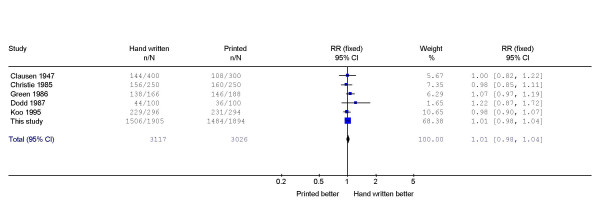
Meta-analysis of randomised controlled trials comparing hand written with printed signature on a covering letter accompanying a questionnaire.

## Pre-publication history

The pre-publication history for this paper can be accessed here:



## References

[B1] Edwards P, Roberts I, Clarke M, DiGuiseppi C, Pratap S, Wentz R, Kwan I (2005). Methods to influence response to postal questionnaires (Cochrane Methodology Review). The Cochrane Library.

[B2] Dodd DK, Markwiese BJ (1987). Survey response rate as a function of personalized signature on cover letter. Journal of Social Psychology.

[B3] Koo MM, Rohan TE (1995). Printed signatures and response rates. Epidemiology.

[B4] Christie SC (1985). An analysis of three different treatments on the response rate of a mail survey. Student Research Report, Department of Marketing, Massey University.

[B5] Clausen JA, Ford RN (1947). Controlling bias in mail questionnaires.

[B6] Green KE, Stager SF (1986). The effects of personalization, sex, locale, and level taught on educators' responses to a mail survey. Journal of Experimental Education.

[B7] Tully L, Gates S, Brocklehurst P, McKenzie-McHarg K, Ayers S (2002). Surgical techniques during caesarean section operations: results of a national survey of practice in the UK. European Journal of Obstetrics and Gynecology and Reproductive Biology.

